# The Real-World Clinical Outcomes of Heavily Pretreated HER2+ and HER2-Low Metastatic Breast Cancer Patients Treated with Trastuzumab Deruxtecan at a Single Centre

**DOI:** 10.3390/curroncol32010001

**Published:** 2024-12-24

**Authors:** Anna-Maria Lazaratos, Matthew Dankner, Aalya Hamouda, Soumaya Labidi, Victor Cohen, Lawrence Panasci, Jennifer E. Friedmann, François Patenaude, Cristiano Ferrario, Mark Basik, April A. N. Rose, Parvaneh Fallah

**Affiliations:** 1 Rosalind and Morris Goodman Cancer Institute, Montreal, QC H3A 1A3, Canada; anna-maria.lazaratos@mail.mcgill.ca (A.-M.L.); matthew.dankner@mail.mcgill.ca (M.D.); 2 Faculty of Medicine, McGill University, Montreal, QC H3G 2M1, Canada; aalya.hamouda@mail.mcgill.ca; 3 Jewish General Hospital, Montreal, QC H3T 1E2, Canada; soumaya.labidi@mail.mcgill.ca (S.L.); victor.cohen.med@ssss.gouv.qc.ca (V.C.); lawrence.panasci@mcgill.ca (L.P.); jennifer.friedmann.med@ssss.gouv.qc.ca (J.E.F.); francois.patenaude@mcgill.ca (F.P.); cristiano.ferrario@mcgill.ca (C.F.); mark.basik@mcgill.ca (M.B.); april.rose@mcgill.ca (A.A.N.R.)

**Keywords:** trastuzumab, deruxtecan, TDXd, antibody–drug conjugate, breast cancer

## Abstract

Background: Trastuzumab deruxtecan (TDXd) is an antibody–drug conjugate that has demonstrated impressive activity in randomized controlled clinical trials in the context of patients with HER2-amplified and HER2-low metastatic breast cancer. We aimed to review the activity and adverse event profile of TDXd in heavily pretreated breast cancer patients in real practice. Methods: We describe a single-center retrospective case series of metastatic breast cancer patients who were treated with TDXd. The outcomes of interest were the overall response rate, overall survival, progression-free survival and grade 4–5 adverse events. Objective responses and PFS were assessed in accordance with RECIST 1.1 criteria. Results: We identified 38 patients treated with TDXd. Of these, 15 patients had classically defined HER2-positive (HER2+) breast cancer, 4 of whom had active central nervous system (CNS) metastases. A total of 23 patients had HER2-low breast cancer, 2 of whom had active CNS disease. Of the 33 patients evaluable for response, 21 (63%) patients had a response to treatment, including three (9%) complete responses. Outcomes were similar between patients with a HER2+ and HER2-low status, as well as in patients with or without CNS metastases. No patients experienced grade 4 or 5 toxicities, and four of thirty-eight patients (10.5%) experienced pneumonitis (two patients with grade 3 pneumonitis, one patient with grade 2 and one patient with grade 1), resulting in TDXd discontinuation for three patients (with steroid administration in two patients). Conclusions: TDXd demonstrates impressive activity with manageable adverse event profiles in this heavily pretreated population that includes patients with active CNS metastases.

## 1. Introduction

In recent years, the treatment landscape for metastatic breast cancer has been transformed by the development of antibody–drug conjugates, which offer a more tailored approach to combatting tumor growth and proliferation. Among these, trastuzumab deruxtecan (TDXd) represents a significant advancement, particularly for HER2-positive and, most recently, HER2-low-expressing cancers. Despite the proven efficacy of earlier treatments such as trastuzumab and pertuzumab in HER2-positive cancers, a substantial number of patients continue to exhibit disease progression, particularly in the CNS, underscoring the need for novel therapeutic options for this patient population. TDXd is an antibody–drug conjugate (ADC) that consists of an anti-human epidermal growth factor receptor 2 (HER2) monoclonal antibody (trastuzumab), a cleavable linker and a cytotoxic payload (deruxtecan, a topoisomerase 1 inhibitor) [1]. TDXd has demonstrated impressive efficacy in the context of HER2-positive (HER2+) and HER2-low breast cancers [2,3,4,5,6,7], including in patients with central nervous system (CNS) metastases [8,9,10,11,12,13], as well as with other HER2-expressing or mutated tumor types [14,15,16,17,18,19,20,21,22,23].

While the demonstrated efficacy of TDXd has led to its implementation in clinical practice broadly for patients with metastatic HER2+ or HER2-low breast cancer, it has been associated with important toxicities that require close observation. These include the cardiotoxicities traditionally associated with trastuzumab and a small but meaningful percentage of patients who experience interstitial lung disease/pneumonitis as a potentially fatal severe adverse event. Previous trials have demonstrated a 10–15% rate of pneumonitis and a 1–2% rate of grade 5 pneumonitis [24,25,26,27]. For this reason, real-world data documenting the toxicities of TDXd outside of carefully selected clinical trial populations are worthwhile. The DESTINY-Breast 01-04 trials that studied TDXd in HER2+ and HER2-low breast cancers employed inclusion and exclusion criteria that excluded patients with active central nervous system metastases [2,4,5,7]. The DESTINY-Breast12 study included patients with active parenchymal brain metastases, though excluded patients who received greater than two lines of prior therapy in the metastatic setting, had an Eastern Cooperative Oncology Group performance status (ECOG PS) of two or greater, or who had leptomeningeal metastases [28]. Therefore, real-world data describing the efficacy of TDXd in heavily pretreated patients with an active CNS metastasis are important. The aim of our study was to evaluate the real-world clinical outcomes of TDXd in heavily pretreated patients with HER2-positive and HER2-low metastatic breast cancer. Herein, we present the results of a case series of 38 metastatic breast cancer patients treated with TDXd (including 15 with CNS involvement (39.5%)) to document responses to therapy and adverse events related to TDXd treatment. Our work provides insights into TDXd’s activity and adverse event profile outside the controlled conditions of clinical trials, particularly in a population that has been previously treated with multiple lines of therapy.

## 2. Methods

Included patients met the following inclusion criteria: a diagnosis of metastatic breast cancer and treatment with TDXd outside of a clinical trial setting and as part of a compassionate access program or funded by private insurance at the Jewish General Hospital in Montreal, Quebec, Canada. Treatment was initiated between September 2021 and November 2023. Patients with HER2+ breast cancer were defined as having HER2 3+ immunohistochemistry (IHC) staining or HER2 2+ IHC and positive fluorescence in situ hybridization (FISH). HER2-low breast cancer was defined as HER2 2+ IHC with FISH negativity, or HER2 1+ IHC. The primary endpoints of interest were the overall response rate (ORR) defined using RECIST 1.1 criteria [29] and grade 4–5 adverse events graded based on the common terminology criteria version 5 for adverse events [30]. Secondary endpoints included progression-free survival (PFS), the overall survival (OS) and grade 1–3 adverse events. Active CNS metastases were defined as newly diagnosed brain metastases that had received no prior local therapy or brain metastases progressing after previous local therapy.

Patient charts were accessed for inclusion in this study without informed consent as part of a CIUSSS West-Central Montreal Research Ethics Board-approved protocol (Protocol Number 2024-3895), in an anonymized fashion, in concordance with the Declaration of Helsinki. The data cut-off was 11 January 2024.

Statistical analyses and the creation of graphics were performed with Graphpad PRISM version 7 and STATA version 18. Statistical analyses were conducted using R statistical software version 4.0.3. Continuous variables were summarized using medians and interquartile ranges and categorical variables using frequencies and percentages. A Kaplan–Meier survival analysis was employed to estimate the overall survival and progression-free survival. Multivariable Cox regression models were used to adjust for potential confounders, including age, prior treatments and the hormone receptor status. A *p*-value of <0.05 was considered statistically significant.

## 3. Results

### 3.1. Patient Characteristics

We identified a total of 38 patients with metastatic breast cancer who were treated with TDXd (Table 1, Supplemental Figure S1). The median age was 57 years old (range of 33–76). A total of 15 patients were HER2-positive (39.5%), and 23 patients were HER2-low (60.5%) (Table 1). One of these twenty-three HER2-low patients had an IHC score of zero in the sampled metastatic lesion, but an IHC score of two+ in the primary tumor. Of the 15 HER2-positive patients, 4 had active central nervous system (CNS) disease (26.7%). Twenty of twenty-three HER2-low patients were estrogen receptor (ER)-positive (87%), and two had active CNS metastases (8.7%). Patients received a median of four lines of therapy prior to the initiation of TDXd (range 1–12). At the time of TDXd initiation, 15 patients were known for CNS metastases (39.5%) (6 patients with active CNS disease at the time of TDXd initiation (15.8%)), 19 had lung metastases (50%), 18 had liver metastases (47.4%) and 26 had bone metastases (68.4%).

### 3.2. Responses to TDXd

**Figure 1:**
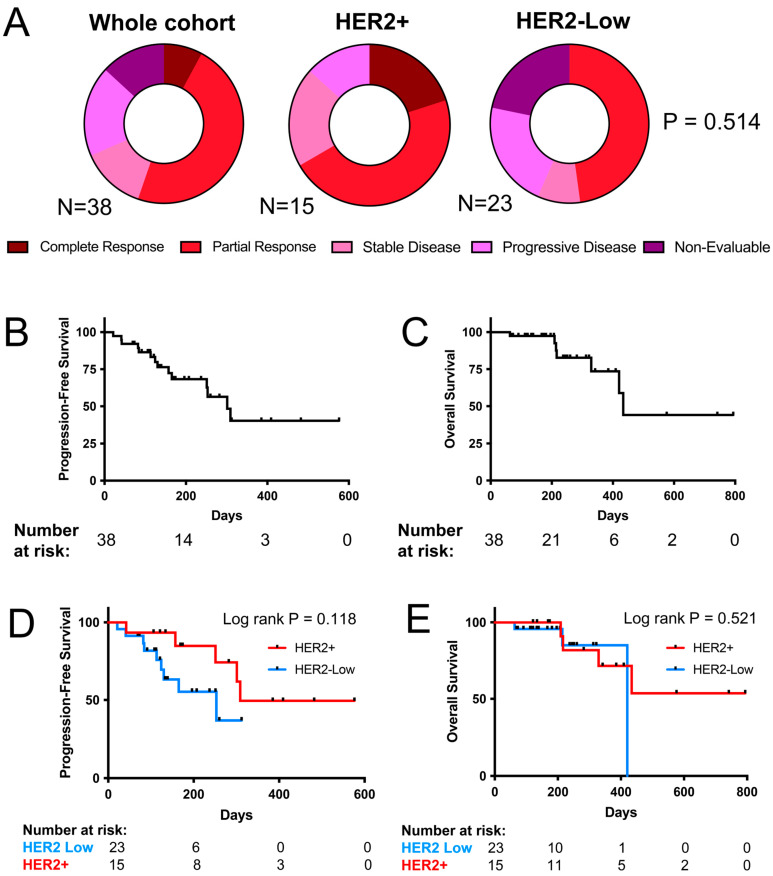
(**A**) The best responses according to RECIST 1.1 criteria [29] per patient in the whole cohort (**left**), HER2+ patients (**middle**) and HER2-low patients (**right**). *p*-value calculated with Fisher’s Exact Test and compared ORR between HER2+ and HER2-low patients. (**B**) The PFS and (**C**) OS in the total patient cohort. (**D**) PFS and (**E**) OS of HER2+ compared to HER2-low patients. *p*-value calculated using log rank test.

A total of 33 patients were evaluable for response (Table 2). Twenty-one of thirty-three patients experienced a RECIST 1.1 response to TDXd (63.6%), including 3 complete responses (9.1%) and 18 partial responses (54.5%) (Figure 1A). Five patients had stable disease as the best response (15.2%), and seven patients had progressive disease on treatment (21.2%). The median follow-up time was 245 days. We observed no significant difference in the ORR in patients with HER2+ compared to HER2-low metastatic breast cancer (Figure 1A). A total of 21 patients remained on TDXd at the data cut-off time point (55.3%), with 14 patients stopping treatment due to progressive disease (36.8%) and 3 patients stopping TDXd due to adverse events (7.9%). The median PFS was 301 days, and the median OS was 434 days (Figure 1B,C). We observed a trend toward prolonged PFS amongst the HER2+ patients that did not reach statistical significance (Figure 1D,E). Clinical variables associated with the PFS and OS are presented in Table 3 and Table 4, respectively.

### 3.3. Responses to TDXd in the CNS Cohort

All 15 patients with CNS metastases at the time of TDXd initiation were treated with local modalities (radiation and/or surgery) prior to initiating TDXd treatment. Of these 15 patients, 10 patients received brain radiotherapy prior to TDXd initiation (66.7%) and 5 patients underwent both surgery and brain radiotherapy for their brain metastases (33.3%) (Table 1). Of 15 patients with treated CNS metastases at the time of TDXd initiation, 11 patients had at least one follow-up brain scan to monitor the CNS response. Six out of fifteen patients had progression of CNS metastases after previous local therapy and prior to initiating TDXd (40%), denoting these as active CNS metastases.

**Figure 2:**
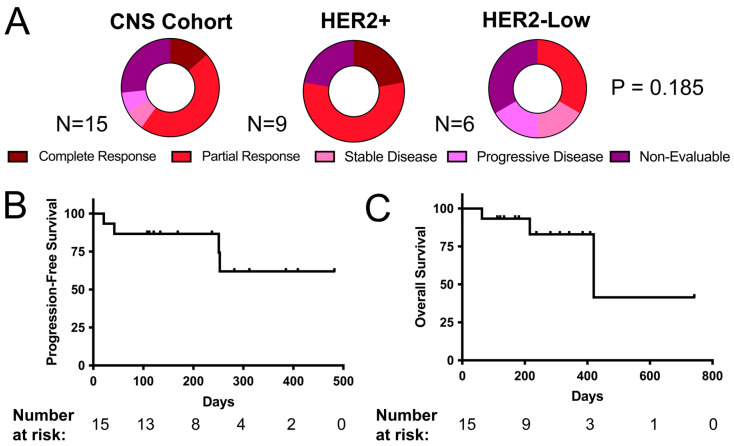
(**A**) The best CNS-specific responses according to RECIST 1.1 criteria [29] per patient in the whole cohort with CNS metastases (**left**), HER2+ patients (**middle**) and HER2-low patients (**right**). (**B**) The PFS and (**C**) OS in the patient cohort with CNS metastases. *p*-value calculated using log rank test. Abbreviations: CNS, central nervous system.

In the entire CNS cohort, out of eleven patients with at least one follow-up brain scan (73.3%), two patients (previously described [12]) had a complete resolution of brain and leptomeningeal lesions (13.3%), seven patients had a partial response in parenchymal brain metastases and leptomeningeal lesions (46.7%), one patient had stable brain metastases in the context of an extracranial partial response (66.7%) and one patient had progressive brain and leptomeningeal metastases (6.7%) (Figure 2A). The response rates of subgroups of patients with either a locally treated or active CNS metastasis prior to starting TDXd are presented in Supplemental Figures S2A and S2B, respectively. We observed no significant difference in the CNS-specific ORR in patients with HER2+ compared to HER2-low metastatic breast cancer with CNS involvement (Supplemental Figure S2A,B). Of the four remaining patients without brain imaging to monitor the CNS-specific response, one patient stopped TDXd 42 days after initiation due to extracranial progressive disease, one patient stopped TDXd after 108 days due to an adverse reaction and two patients remained on TDXd for 112 and 169 days, respectively. Two patients had no CNS metastases at the initiation of TDXd but developed leptomeningeal metastases while undergoing treatment (5.3%). In patients with a CNS metastasis, the median PFS was not reached, and the median OS was 420 days (Figure 2B,C). Among patients with CNS involvement, we observed no significant difference in the PFS or OS in patients with HER2+ compared to HER2-low metastatic breast cancer (Supplemental Figure S2C,D). We also observed no significant difference in the PFS or OS between patients with and without any CNS metastases (Supplemental Figure S2E–J). This observation remained consistent when comparing patients with active CNS disease at the time of TDXd initiation to those without CNS involvement (Supplemental Figure S2K–P), as well as when comparing patients with active versus stable brain metastases (Supplemental Figure S2Q–V). These findings were consistent across all breast cancer patients, as well as within subgroups of HER2-positive breast cancer and HER2-low breast cancer patients (Supplemental Figure S2).

### 3.4. Adverse Events

Twenty-seven of thirty-eight patients treated with TDXd experienced adverse events on treatment (71.1%) (Table 5). No grade 4 or grade 5 toxicities were experienced in the cohort, but four patients had grade 3 toxicities (14.8%). These included two patients with grade 3 fatigue (7.4%), one patient with grade 3 neutropenia (3.7%) and one with grade 3 thrombocytopenia (3.7%). Common grade 1–2 adverse events included fatigue (thirteen patients (48.1%), pneumonitis (four patients experienced pneumonitis, three grade 2 and one grade 1, 14.8%), nausea/vomiting (seventeen patients, 63%), diarrhea (seven patients, 25.9%), alopecia (five patients, 18.5%), neuropathy, likely as residual toxicity from prior chemotherapy (five patients, 18.5%), and weight loss/anorexia (five patients, 18.5%). The three patients in the cohort who stopped treatment due to adverse events did so because of grade 2 pneumonitis (7.9%).

## 4. Discussion

Herein, we describe the experience at our center with a real-world cohort of heavily pretreated patients treated with TDXd outside of a clinical trial setting. Despite the different ORR between HER2+ and HER2-low patients observed in an informal cross-trial comparison of DESTINY-Breast-03 [5] and DESTINY-Breast-04 [4], we observed no significant difference in the PFS (*p* = 0.118) or OS (*p* = 0.521) between the 15 HER2+ and 23 HER2-low breast cancer patients. However, an important limitation of this finding is that our study is underpowered to detect a statistically significant difference at this time. The relatively small sample size may limit the generalizability of our findings. Selection bias is of particular concern, as the patients who participate in a study with a small sample may not be representative of the general population. Larger prospective studies are necessary to validate our results, confirm the study endpoints and ensure the reproducibility of the reported clinical outcomes.

Even though the patients included in the cohort were heavily pretreated, with a median of four lines of prior therapy in the metastatic setting, we observed clinical responses in 21 of 33 patients evaluable for clinical response, with durable responses lasting a median of approximately 1 year. This included a subset of patients who were previously exposed to T-DM1 and would not have been eligible for the DESTINY-Breast 03 trial.

Our cohort also included patients with active brain and leptomeningeal metastases, a population that was not included in the DESTINY-Breast 03 or 04 randomized clinical trials. Indeed, other studies have similarly shown an excellent activity of TDXd in patients with CNS metastases [10,12,13,28]. While the sample size was small, it is noteworthy that patients with CNS metastases in our cohort who derived a clinical benefit from TDXd all received some form of brain radiotherapy. This contrasts with a CNS metastasis-naïve patient in our cohort who progressed with leptomeningeal metastases while on TDXd. It has previously been demonstrated that the presence of CNS lesions and brain radiotherapy plays an important role in increasing the cerebrospinal fluid concentrations of trastuzumab [31]. While TDXd has the potential to exhibit excellent CNS activity, it is possible that brain radiotherapy may be required to further open parts of the blood–tumor barrier to increase drug concentrations [32]. Interestingly, patients with and without a CNS metastasis exhibited similar PFS and OS outcomes. This observation suggests that TDXd is similarly effective in the context of CNS lesions as it is in extracranial metastases, potentially distinguishing TDXd from other HER2-targeted monoclonal antibodies, such as trastuzumab and pertuzumab [33]. Our findings contribute to the growing body of evidence supporting the efficacy of TDXd in diverse patient populations, including those not typically represented in randomized clinical trials. By documenting its effectiveness and manageable safety profile in a real-world setting, our study reinforces the adaptability of TDXd in clinical practice, particularly for heavily pretreated patients and those with CNS metastases. TDXd represents a promising treatment option for this complex patient group, emphasizing the ongoing need for research into personalized therapeutic strategies for metastatic breast cancer.

While we observed an impressive activity of TDXd in our cohort, the toxicities associated with this novel agent are noteworthy. Only 3 of 38 patients who received TDXd stopped treatment due to toxicity. Three patients experienced pneumonitis while on TDXd, an important adverse event that should be managed urgently given the possibility of progression to grade 4 and 5 toxicities demonstrated in trial settings [26]. It is also possible that, with an increased follow-up time, the incidence of pneumonitis/interstitial lung disease in our cohort would increase further, given that only 55% of reported events in the literature occurred within 3 months of treatment, a time point that some patients described herein had yet to reach [26]. This raises the importance of hypervigilance in monitoring patients for signs of lung toxicity while on TDXd.

Mechanistically, lung toxicity associated with TDXd is believed to occur due to the Fc region of the antibody interacting with FcγR on alveolar macrophages, mediating the uptake [34]. This toxicity may therefore be independent of the targeted antigen and may be relevant for further study in other ADCs in development, such as patritumab and datopotamab deruxtecan [35,36]. The impact of TDXd on the quality of life, particularly regarding treatment-related adverse events, such as interstitial lung disease, warrants the extensive monitoring of patients. Our findings indicate that while TDXd is effective, monitoring and managing adverse events are paramount to maintaining patients’ quality of life during treatment. To ensure the safety and efficacy of TDXd, treating oncologists monitor patients for symptoms of lung toxicity through regular appointments, given the known risks of interstitial lung disease associated with this agent. These assessments are conducted at the baseline and generally every 3 weeks at clinic visits prior to each cycle, and by imaging approximately every three–four cycles. Upon the appearance of any symptoms or imaging findings indicating potential complications, oncologists can rapidly intervene to manage these adverse effects, either through the addition of steroids and/or treatment pausing or discontinuation.

This case series represents an early real-world cohort of patients with HER2+ and HER2-low metastatic breast cancer treated with TDXd. Despite the cohort comprising more heavily pretreated patients than the clinical trial populations, including a subset with active CNS metastases, TDXd demonstrated impressive activity. While drug-related toxicities were present, including a number of patients who experienced lung toxicity, this was manageable in a majority of patients with a close follow-up. Our findings are consistent with those reported in other real-world studies and clinical trials, such as the DESTINY-Breast01 trial. However, unlike these studies, our real-world cohort includes a more diverse patient population, offering unique insights into the efficacy and safety of TDXd in heavily pretreated patients. Our study underscores the efficacy of TDXd in heavily pretreated HER2-positive and HER2-low metastatic breast cancer patients, notably in patients with a CNS metastasis. Future research should focus on longitudinal studies to assess the long-term outcomes and further explore the impact of TDXd on different subgroups within the metastatic breast cancer population. To address the limitations observed in this study regarding the statistical power, future research will aim to recruit larger patient cohorts and may incorporate adaptive trial designs. These measures will enhance our ability to detect significant differences across various patient subgroups and provide more definitive conclusions about the efficacy and safety of TDXd.

## Figures and Tables

**Table 1:** Individual patient characteristics.

Variable	Entire Cohort No. (%)	HER2-Low No. (%)	HER2+ No. (%)	*p* (Fisher’s Exact)
**HER2 status**	**38 (100)**	**23 (60.5)**	**15 (39.5)**	
**Patient Characteristics**				
**Age, years**				
<60	22 (57.9)	11 (47.8)	11 (73.3)	0.111
≥60	16 (42.1)	12 (52.2)	4 (26.7)	
**Estrogen receptor status**				
Estrogen receptor-negative	9 (23.7)	3 (13.0)	6 (40.0)	0.065
Estrogen receptor-positive	29 (76.3)	20 (87.0)	9 (60.0)	
**Progesterone receptor status**				
Progesterone receptor-negative	19 (50)	10 (43.5)	9 (60.0)	0.187
Progesterone receptor-positive	18 (47.4)	13 (56.5)	5 (33.3)	
Unknown	1(2.6)	0(0)	1(6.7)	
**Lines of therapy in metastatic setting prior to starting TDXd**				
<4	16 (42.1)	8 (34.8)	8 (53.3)	0.213
≥4	22 (57.9)	15 (65.2)	7 (46.7)	
**Prior trastuzumab**				
No	21 (55.3)	20 (87.0)	1 (6.7)	**<0.001**
Yes	17 (44.7)	3 (13.0)	14 (93.3)	
**Prior T-DM1**				
No	31 (81.6)	23 (100)	8 (53.3)	**0.001**
Yes	7 (18.4)	0 (0)	7 (46.7)	
**Prior HER2 TKI**				
No	31 (81.6)	22 (95.7)	9 (60)	**0.01**
Yes	7 (18.4)	1 (4.3)	6 (40)	
**All with CNS involvement at time of starting TDXd**				
No	23 (60.5)	17 (73.9)	6 (40)	0.04
Yes	15 (39.5)	6 (26.1)	9 (60)	
**Treated/stable versus active CNS involvement at time of starting TDXd**				
Treated/stable	9 (60)	4 (66.7)	5 (55.6)	0.545
Active	6 (40)	2 (33.3)	4 (44.4)	
**Treatment for CNS involvement**				
Radiation	9 (60)	5 (83.3)	4 (44.4)	0.168
Both surgery and radiation	6 (40)	1 (16.7)	5 (55.6)	

Abbreviations: TDXd, trastuzumab deruxtecan; T-DM1, trastuzumab emtansine; TKI, tyrosine kinase inhibitor; CNS, central nervous system. Bold values indicate *p* < 0.05.

**Table 2:** Individual patient outcomes.

Variable	Entire Cohort No. (%)	HER2-Low No. (%)	HER2+ No. (%)	*p* (Fisher’s Exact)	Pearson’s χ2
**HER2 status**	**38 (100)**	**23 (60.5)**	**15 (39.5)**		
**TDXd Treatment Characteristics**					
**Treatment response according to RECIST1.1**					
Progressive disease	7 (18.4)	5 (21.7)	2 (13.3)	0.283	5.144, *p* = 0.162
Stable disease	5 (13.2)	2 (8.7)	3 (20)	0.409	
Partial response	18 (47.4)	13 (56.5)	5 (33.3)	0.187	
Complete response	3 (7.9)	0 (0)	3 (20)	0.083	
**CNS-specific treatment response in all patients with CNS involvement (15 patients) according to RECIST1.1**					
Progressive disease	1 (6.7)	1 (16.7)	0 (0)	0.375	4.827, *p* = 0.185
Stable disease	1 (6.7)	1 (16.7)	0 (0)	0.375	
Partial response	7 (46.7)	2 (33.3)	5 (55.6)	0.47	
Complete response	2 (13.3)	0 (0)	2 (22.2)	0.375	
**CNS-specific treatment response in patients with treated/stable (9 patients; 4 HER2-Low, 5 HER2+) CNS involvement according to RECIST1.1**					
Progressive disease	0 (0)	0 (0)	0 (0)	NA	2.625, *p* = 0.269
Stable disease	1 (11.1)	1 (25)	0 (0)	0.5	
Partial response	4 (44.4)	1 (25)	3 (60)	0.6	
Complete response	1 (11.1)	0 (0)	1 (20)	0.5	
**CNS-specific treatment response with active CNS involvement (6 patients; 2 HER2-Low, 4 HER2+) according to RECIST1.1**					
Progressive disease	1 (16.7)	1 (50)	0 (0)	0.25	2.222, *p* = 0.329
Stable disease	0 (0)	0 (0)	0 (0)	NA	
Partial response	3 (50)	1 (50)	2 (50)	0.7	
Complete response	1 (16.7)	0 (0)	1 (25)	0.75	

Abbreviations: TDXd, trastuzumab deruxtecan; CNS, central nervous system; NA, not applicable. Bold values indicate *p* < 0.05.

**Table 3:** Progression-free survival rates associated with clinical variables.

Characteristics	Patients	Median PFS (Days)	Univariable Hazard Ratio	Univariable 95% CI	Univariable *p*-Value
Entire cohort	38	301			
**Study Characteristics**					
**Age, years**					
<60	22	NA	3.186	1.017–9.985	**0.047**
≥60	16	251			
**Estrogen receptor status**					
Estrogen receptor-negative	9	301	0.837	0.262–2.681	0.765
Estrogen receptor-positive	29	309			
**Progesterone receptor status**					
Progesterone receptor-negative	19	309	3.127	0.925–10.565	0.066
Progesterone receptor-positive	18	251			
Unknown	1	NA			
**Breast cancer at time of diagnosis**					
Local	27	309	0.915	0.300–2.794	0.876
Metastatic	11	301			
**Breast cancer features**					
Ductal	28	NA	0.365	0.109–1.218	0.101
Lobular	4	157	3.03	0.618–14.866	0.172
Both	3	253	2.046	0.445–9.416	0.358
Unknown	3	301	0.688	0.088–5.352	0.721
**Breast cancer grade**					
1	1	NA	5.25E+16	0–NA	1
2	12	309	0.822	0.231–2.924	0.762
3	16	NA	0.821	0.237–2.844	0.755
Unknown	9	301	1.615	0.495–5.266	0.427
**Size of primary breast tumor (if primary breast cancer at time of diagnosis)**					
<5 cm	10	NA	1.918	0.334–11.019	0.465
≥5 cm	8	253			
**Nodal involvement (if primary breast cancer at time of diagnosis)**					
No	7	NA	0.667	0.165–2.703	0.571
Yes	20	309			
**Neoadjuvant treatment (if primary breast cancer at time of diagnosis)**					
No	15	NA	1.762	0.427–7.264	0.433
Yes	14	253			
**Adjuvant treatment (if primary breast cancer at time of diagnosis)**					
No	3	NA	0.592	0.073–4.834	0.625
Yes	26	309			
**Adjuvant breast radiation (if primary breast cancer at time of diagnosis)**					
No	9	251	0.833	0.250–2.779	0.766
Yes	26	309			
**Mutational burden**					
BRCA1/2	4	253	0.701	0.141–3.482	0.664
PIK3CA	7	251	2.483	0.607–10.164	0.206
CHEK2	2	253	0.688	0.124–3.817	0.669
ESR1	3	253	0.773	0.145–4.120	0.763
TP53	2	84	4.502	0.821–24.696	0.083
**Lines of therapy in metastatic setting prior to starting TDXd**					
<4	16	NA	1.166	0.389–3.496	0.784
≥4	22	301			
**Prior trastuzumab**					
No	21	251	0.683	0.228–2.052	0.497
Yes	17	309			
**Prior T-DM1**					
No	31	253	0.659	0.176–2.459	0.535
Yes	7	309			
**Prior HER2 TKI**					
No	31	301	0.691	0.188–2.544	0.579
Yes	7	309			
**Extracranial metastasis at time of starting TDXd**					
Liver	18	253	2.121	0.708–6.358	0.179
Lung	19	301	1.347	0.463–3.919	0.584
Bone	26	301	1.662	0.457–6.041	0.44
Skin	5	82	5.693	1.426–22.734	**0.014**
Chest wall	3	NA	2.396	0.277–20.741	0.428
Adrenal	5	309	0.562	0.122–2.597	0.461
Pleural	5	NA	1.528	0.334–6.988	0.585
Lymph node	27	301	0.861	0.268–2.762	0.801
**All with CNS involvement at time of starting TDXd**					
No	23	301	0.434	0.134–1.410	0.165
Yes	15	NA			
**Treated/stable vs. active CNS involvement at time of starting TDXd**					
Treated/stable	9	NA	0.634	0.065–6.134	0.694
Active	6	NA			
**Treatment for CNS involvement**					
Radiation	9	NA	2.76	0.283–26.954	0.383
Radiation and surgery	6	NA	0.362	0.037–3.539	0.383
**Treatment Characteristics**					
**Treatment response according to RECIST1.1**					
Progressive disease	7	130	13.289	3.240–54.504	**<0.001**
Stable disease	5	251	0.431	0.056–3.320	0.419
Partial response	18	309	0.579	0.194–1.729	0.327
Complete response	3	NA	4.43E-16	0–NA	1
**CNS-specific treatment response according to RECIST1.1**					
Progressive disease	1	NA	9.25 × 10^16^	NA	NA
Stable disease	1	NA	5.292	0.322–86.928	0.243
Partial response	7	NA	0.374	0.033–4.179	0.424
Complete response	2	NA	4.23 × 10^−17^	0–NA	1
**Adverse events**					
No	11	NA	0.331	0.091–1.206	0.094
Yes	27	309			
**Adverse event grades**					
Grades 1–2	25	309	0.375	0.101–1.388	0.142
Grade 3	6	253	1.407	0.386–5.127	0.605
**All adverse event grades**					
Fatigue	15	309	0.299	0.082–1.087	0.067
Neuropathy	5	NA	0.193	0.024–1.542	0.121
Alopecia	5	301	0.929	0.250–3.453	0.913
Nausea/vomiting	17	309	0.434	0.146–1.295	0.135
Diarrhea	7	301	0.949	0.264–3.419	0.937
Shortness of breath/cough	3	NA	6.10 × 10^−17^	0–NA	1
Anorexia	5	309	0.667	0.147–3.017	0.599
Anemia	2	NA	1.56 × 10^−15^	0–NA	1
Neutropenia	4	157	2.631	0.719–9.619	0.144
Thrombocytopenia	2	113	4.29	0.908–20.274	0.066
Pneumonitis	4	NA	6.09 × 10^−17^	0–NA	1
**TDXd changes due to adverse events**					
Delayed	14	301	2.223	0.266–18.579	0.461
Dose reduced	12	309	0.819	0.155–4.331	0.814
Stopped	3	NA	5.33 × 10^−16^	0–NA	1

Abbreviations: PFS, progression-free survival; CI, confidence interval; TDXd, trastuzumab deruxtecan; T-DM1, trastuzumab emtansine; TKI, tyrosine kinase inhibitor; CNS, central nervous system; NA, not applicable; cm, centimeter. Bold values indicate *p* < 0.05.

**Table 4:** Overall survival rates associated with clinical variables.

Characteristics	Patients	Median OS (Days)	Univariable Hazard Ratio	Univariable 95% CI	Univariable *p*-Value
Entire cohort	38	434			
**Study Characteristics**					
**Age, years**					
<60	22	420	0.987	0.219–4.444	0.986
≥60	16	434			
**Estrogen receptor status**					
Estrogen receptor-negative	9	434	1.239	0.234–6.570	0.801
Estrogen receptor-positive	29	420			
**Progesterone receptor status**					
Progesterone receptor-negative	19	434	1.163	0.257–5.266	0.845
Progesterone receptor-positive	18	420			
Unknown	1	NA			
**Breast cancer at time of diagnosis**					
Local	27	420	0.561	0.108–2.922	0.493
Metastatic	11	434			
**Breast cancer features**					
Ductal	28	NA	0.152	0.028–0.828	0.029
Lobular	4	209	11.136	0.665–186.343	0.094
Both	3	420	4.097	0.668–25.123	0.127
Unknown	3	434	0.496	0.055–4.497	0.533
**Breast cancer grade**					
1	1	NA	5.25 × 10^16^	0–NA	1
2	12	420	1.067	0.144–7.922	0.949
3	16	NA	0.312	0.032–3.083	0.319
Unknown	9	434	1.61	0.350–7.418	0.541
**Size of primary breast tumor (if primary breast cancer at time of diagnosis)**					
<5 cm	10	NA	4.73 × 10^15^	0–NA	1
≥5 cm	8	420			
**Nodal involvement (if primary breast cancer at time of diagnosis)**					
No	7	NA	2.46 × 10^15^	0–NA	1
Yes	20	420			
**Neoadjuvant treatment (if primary breast cancer at time of diagnosis)**					
No	15	NA	3.40 × 10^15^	0–NA	1
Yes	14	329			
**Adjuvant treatment (if primary breast cancer at time of diagnosis)**					
No	3	NA	0.291	0.030–2.808	0.286
Yes	26	420			
**Adjuvant breast radiation (if primary breast cancer at time of diagnosis)**					
No	9	NA	2.416	0.280–20.848	0.422
Yes	26	420			
**Genomic characteristics**					
BRCA1/2 mutation	4	420	1.192	0.154–9.235	0.866
PIK3CA mutation	7	434	1.16 × 10^−17^	0–NA	1
CHEK2 mutation	2	420	1.304	1.161–10.537	0.803
ESR1 mutation	3	420	0.951	0.085–10.587	0.968
TP53 mutation	2	214	1.93 × 10^16^	0–NA	1
**Lines of therapy in metastatic setting prior to starting TDXd**					
<4	16	NA	0.671	0.147–3.070	0.607
≥4	22	434			
**Prior trastuzumab**					
No	21	NA	0.889	0.136–5.829	0.903
Yes	17	434			
**Prior T-DM1**					
No	31	420	0.711	0.107–4.731	0.725
Yes	7	434			
**Prior HER2 TKI**					
No	31	434	1.49	0.305–7.288	0.622
Yes	7	420			
**Extracranial metastasis at time of starting TDXd**					
Liver	18	434	1.045	0.231–4.721	0.954
Lung	19	434	1.542	0.340–6.985	0.574
Bone	26	420	2.833	0.330–24.322	0.342
Skin	5	216	14.103	1.900–104.705	**0.01**
Chest wall	3	NA	4.09 × 10^−15^	0–NA	1
Adrenal	5	434	0.893	0.166–4.820	0.896
Pleural	5	NA	2.00 × 10^−16^	0–NA	1
Lymph node	27	434	2.454	0.294–20.502	0.407
**CNS involvement at time of starting TDXd**					
No	23	434	0.986	0.215–4.518	0.985
Yes	15	420			
**Treated/stable vs. active CNS involvement at time of starting TDXd**					
Treated/stable	9	420	0.712	0.063–8.022	0.783
Active	6	NA			
**Treatment for CNS involvement**					
Radiation	9	420	4.43 × 10^15^	0–NA	1
Radiation and surgery	6	NA	1.12 × 10^−17^	0–NA	1
**Treatment Characteristics**					
**Treatment response according to RECIST1.1**					
Progressive disease	7	214	1.28 × 10^16^	0–NA	1
Stable disease	5	NA	4.80 × 10^−16^	0–NA	1
Partial response	18	420	0.829	0.179–3.836	0.81
Complete response	3	NA	6.06 × 10^−17^	0–NA	1
**CNS-specific treatment response according to RECIST1.1**					
Progressive disease	1	NA	3.20 × 10^16^	0–NA	1
Stable disease	1	NA	2.646	0.118–59.102	0.539
Partial response	7	NA	1.25 × 10^−17^	0-NA	1
Complete response	2	NA	1.24 × 10^−17^	0-NA	1
**Adverse events**					
No	11	216	0.086	0.010–0.716	**0.023**
Yes	27	434			
**Adverse event grades**					
Grades 1–2	25	434	0.09	0.011–0.768	**0.028**
Grade 3	6	434	0.375	0.101–1.388	0.142
**All adverse event grades**					
Fatigue	15	434	0.285	0.059–1.378	0.118
Neuropathy	5	434	0.395	0.045–3.449	0.401
Alopecia	5	434	0.711	0.127–3.975	0.698
Nausea/vomiting	17	NA	0.537	0.118–2.437	0.421
Diarrhea	7	329	1.482	0.287–7.655	0.639
Shortness of breath/cough	3	NA	1.14 × 10^−16^	0–NA	1
Anorexia	5	329	0.84	0.092–7.628	0.877
Anemia	2	NA	1.74 × 10^−16^	0–NA	1
Neutropenia	4	434	2.368	0.477–12.548	0.311
Thrombocytopenia	2	214	4	0.406–39.436	0.235
Pneumonitis	4	NA	1.14 × 10^−16^	0–NA	1
**TDXd changes due to adverse events**					
Delayed	14	420	9.67 × 10^15^	0–NA	1
Dose reduced	12	420	2.574	0.284–23.323	0.401
Stopped	3	NA	1.16 × 10^−16^	0–NA	1

Abbreviations: OS, overall survival; CI, confidence interval; TDXd, trastuzumab deruxtecan; T-DM1, trastuzumab emtansine; TKI, tyrosine kinase inhibitor; CNS, central nervous system; NA, not applicable; cm, centimeter. Bold values indicate *p* < 0.05.

**Table 5:** Individual patient adverse events.

Variable	Entire Cohort No. (%)	HER2-Low No. (%)	HER2+ No. (%)	*p* (Fisher’s Exact)
**HER2 status**	**38 (100)**	**23 (60.5)**	**15 (39.5)**	
**Adverse Events to TDXd**				
**Adverse events**				
No	11 (28.9)	9 (39.1)	2 (13.3)	0.087
Yes	27 (71.1)	14 (60.9)	13 (86.7)	
**Grades**				
Grades 1–2	25 (92.6)	12 (85.7)	13 (100)	**0.03**
Grade 3	6 (15.8)	4 (17.4)	2 (13.3)	0.556
**All grades**				
Fatigue	15 (55.6)	6 (42.9)	9 (69.2)	**0.04**
Neuropathy	5 (18.5)	1 (7.1)	4 (30.8)	0.069
Alopecia	5 (18.5)	2 (14.3)	3 (23.1)	0.298
Nausea/vomiting	17 (63.0)	8 (57.1)	9 (69.2)	0.116
Diarrhea	7 (25.9)	3 (21.4)	4 (30.8)	0.261
Shortness of breath/cough	3 (11.1)	0 (0)	3 (23.1)	0.054
Anorexia	5 (18.5)	3 (21.4)	2 (15.4)	0.668
Anemia	2 (7.4)	1 (7.1)	1 (7.7)	0.64
Neutropenia	4 (14.8)	2 (14.3)	2 (15.4)	0.52
Thrombocytopenia	2 (7.4)	2 (14.3)	0 (0)	0.36
Pneumonitis	4 (14.8)	1 (7.1)	3 (23.1)	0.16
**TDXd changes due to adverse events**				
Delayed	14 (51.9)	5 (35.7)	9 (69.2)	0.455
Dose reduced	12 (44.4)	6 (42.9)	6 (46.2)	0.26
Stopped	3 (11.1)	2 (14.3)	1 (7.7)	0.356

Abbreviations: TDXd, trastuzumab deruxtecan. Bold values indicate *p* < 0.05.

## Data Availability

Data will be made available upon request and subject to review and approval by the CIUSSS West-Central Montreal Research Ethics Board.

## Supplementary Materials

The following supporting information can be downloaded at https://www.mdpi.com/article/10.3390/curroncol32010001/s1: Figure S1: A swimmer plot of patients included in the cohort. Purple bars represent time on trastuzumab deruxtecan (TDXd). Arrows represent ongoing treatment. Red stop bars represent the death of the patient. Figure S2: (A) The best CNS-specific responses according to RECIST 1.1 criteria [29] per patient in the whole cohort with central nervous system (CNS) metastases (left), HER2+ patients (middle) and HER2-low patients (right) with treated/stable CNS involvement. (B) The best CNS-specific responses according to RECIST 1.1 criteria [29] per patient in the whole cohort with CNS metastases (left), HER2+ patients (middle) and HER2-low patients (right) with active CNS involvement. *p*-value calculated with Fisher’s Exact Test and Pearson’s chi-squared test and compared overall response rate between HER2+ and HER2-low patients with CNS metastases. (C) PFS and (D) OS of HER2+ compared to HER2-low patients. (E) OS and (F) PFS of patients with and without CNS involvement who received TDXd. (G) OS and (H) PFS of HER2-low patients with and without CNS involvement who received TDXd. (I) OS and (J) PFS of HER2+ patients with and without CNS involvement who received TDXd. (K) OS and (L) PFS of patients with and without active CNS involvement who received TDXd. (M) OS and (N) PFS of HER2-low patients with and without active CNS involvement who received TDXd. (O) OS and (P) PFS of HER2+ patients with and without active CNS involvement who received TDXd. (Q) OS and (R) PFS of patients with stable or active CNS involvement who received TDXd. (S) OS and (T) PFS of HER2-low patients with stable or active CNS involvement who received TDXd. (U) OS and (V) PFS of HER2+ patients with stable or active CNS involvement who received TDXd. *p*-value calculated using log rank test. Abbreviations: CNS, central nervous system; PFS, progression-free survival; OS, overall survival.
