# Supplementary material for: The Real-World Clinical Outcomes of Heavily Pretreated HER2+ and HER2-Low Metastatic Breast Cancer Patients Treated with Trastuzumab Deruxtecan at a Single Centre
Source: Curr Oncol. 2024 Dec 24;32(1):1. doi: 10.3390/curroncol32010001 (PMC11763754; doi:10.3390/curroncol32010001)
Supplement: Supplementary file 1 [file curroncol-32-00001-s001.zip › Supplemental Figure S1.pptx]

## Slide 1
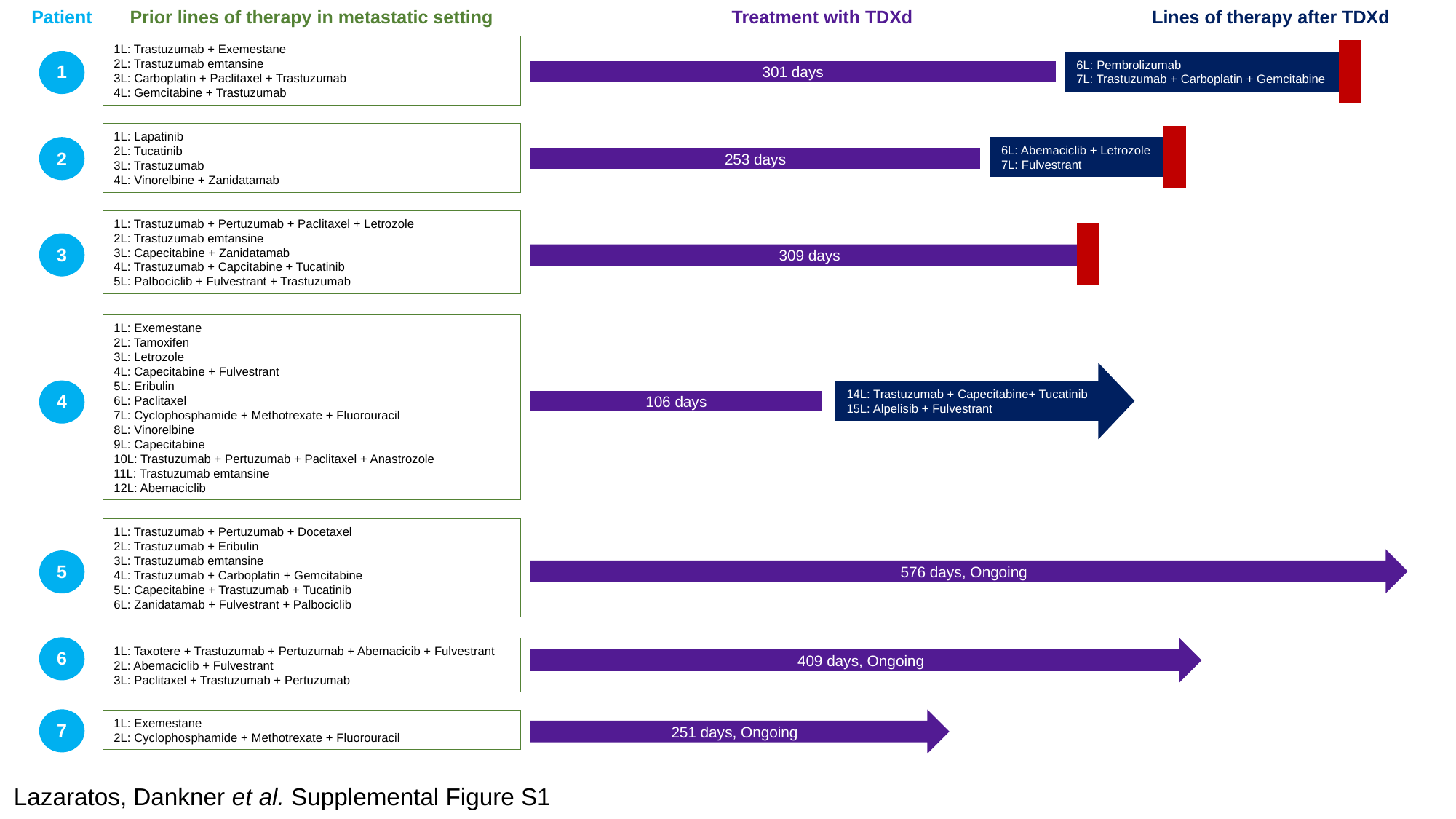

Patient
Prior lines of therapy in metastatic setting
Treatment with TDXd
Lines of therapy after TDXd
1L: Trastuzumab + Exemestane
2L: Trastuzumab emtansine
3L: Carboplatin + Paclitaxel + Trastuzumab
4L: Gemcitabine + Trastuzumab
6L: Pembrolizumab
7L: Trastuzumab + Carboplatin + Gemcitabine
1
301 days
1L: Lapatinib
2L: Tucatinib
3L: Trastuzumab
4L: Vinorelbine + Zanidatamab
6L: Abemaciclib + Letrozole
7L: Fulvestrant
2
253 days
1L: Trastuzumab + Pertuzumab + Paclitaxel + Letrozole
2L: Trastuzumab emtansine
3L: Capecitabine + Zanidatamab
4L: Trastuzumab + Capcitabine + Tucatinib
5L: Palbociclib + Fulvestrant + Trastuzumab
309 days
3
1L: Exemestane
2L: Tamoxifen
3L: Letrozole
4L: Capecitabine + Fulvestrant
5L: Eribulin
6L: Paclitaxel
7L: Cyclophosphamide + Methotrexate + Fluorouracil
8L: Vinorelbine
9L: Capecitabine
10L: Trastuzumab + Pertuzumab + Paclitaxel + Anastrozole
11L: Trastuzumab emtansine
12L: Abemaciclib
14L: Trastuzumab + Capecitabine+ Tucatinib
15L: Alpelisib + Fulvestrant
4
106 days
1L: Trastuzumab + Pertuzumab + Docetaxel
2L: Trastuzumab + Eribulin
3L: Trastuzumab emtansine
4L: Trastuzumab + Carboplatin + Gemcitabine
5L: Capecitabine + Trastuzumab + Tucatinib
6L: Zanidatamab + Fulvestrant + Palbociclib
576 days, Ongoing
5
1L: Taxotere + Trastuzumab + Pertuzumab + Abemacicib + Fulvestrant
2L: Abemaciclib + Fulvestrant
3L: Paclitaxel + Trastuzumab + Pertuzumab
409 days, Ongoing
6
1L: Exemestane
2L: Cyclophosphamide + Methotrexate + Fluorouracil
251 days, Ongoing
7
Lazaratos, Dankner et al. Supplemental Figure S1

## Slide 2
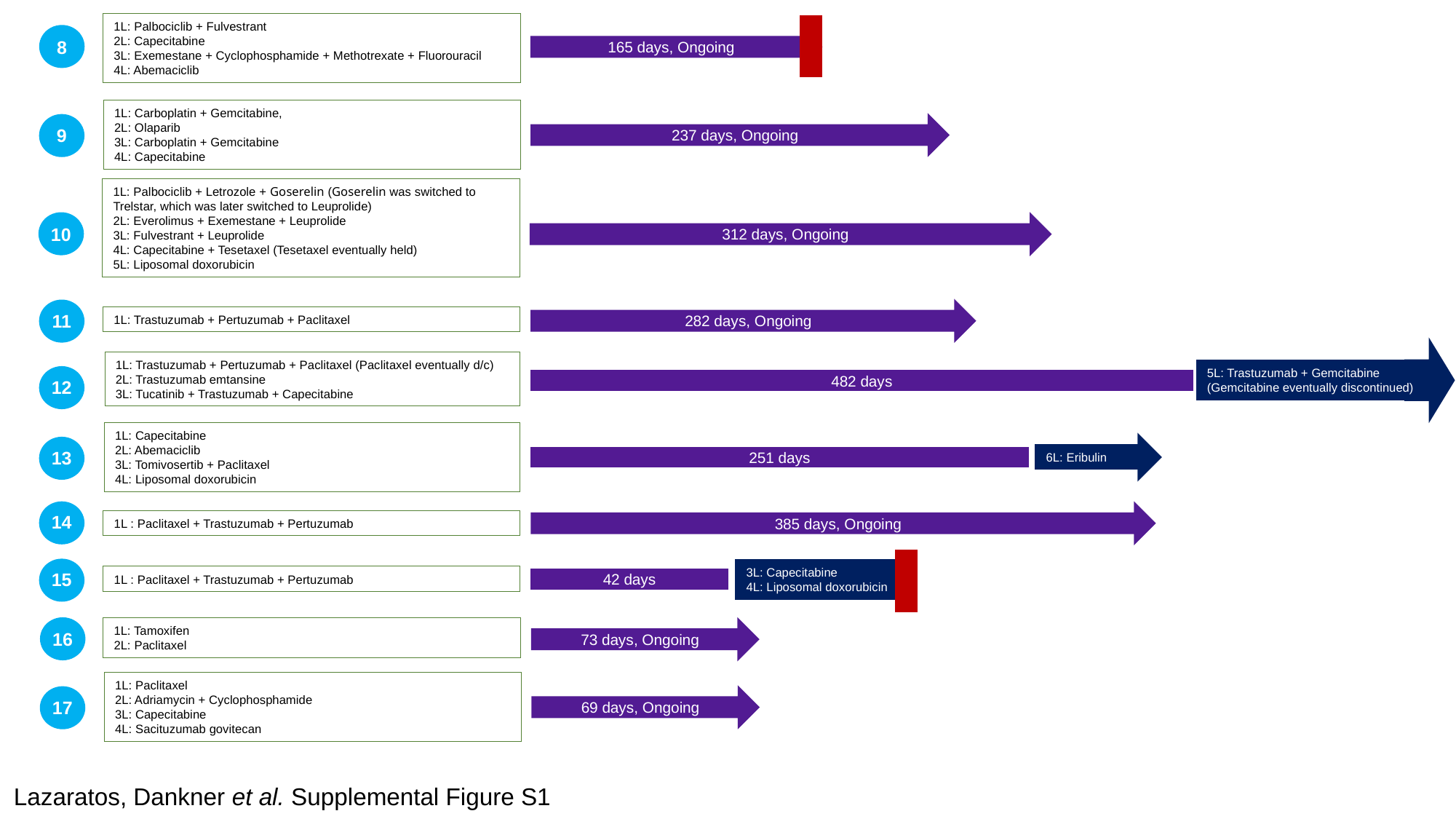

1L: Palbociclib + Fulvestrant
2L: Capecitabine
3L: Exemestane + Cyclophosphamide + Methotrexate + Fluorouracil
4L: Abemaciclib
165 days, Ongoing
8
1L: Carboplatin + Gemcitabine,
2L: Olaparib
3L: Carboplatin + Gemcitabine
4L: Capecitabine
237 days, Ongoing
9
1L: Palbociclib + Letrozole + Goserelin (Goserelin was switched to Trelstar, which was later switched to Leuprolide)
2L: Everolimus + Exemestane + Leuprolide
3L: Fulvestrant + Leuprolide
4L: Capecitabine + Tesetaxel (Tesetaxel eventually held)
5L: Liposomal doxorubicin
312 days, Ongoing
10
282 days, Ongoing
11
1L: Trastuzumab + Pertuzumab + Paclitaxel
1L: Trastuzumab + Pertuzumab + Paclitaxel (Paclitaxel eventually d/c)
2L: Trastuzumab emtansine
3L: Tucatinib + Trastuzumab + Capecitabine
5L: Trastuzumab + Gemcitabine (Gemcitabine eventually discontinued)
12
482 days
1L: Capecitabine
2L: Abemaciclib
3L: Tomivosertib + Paclitaxel
4L: Liposomal doxorubicin
13
6L: Eribulin
251 days
385 days, Ongoing
14
1L : Paclitaxel + Trastuzumab + Pertuzumab
3L: Capecitabine
4L: Liposomal doxorubicin
15
1L : Paclitaxel + Trastuzumab + Pertuzumab
42 days
1L: Tamoxifen
2L: Paclitaxel
73 days, Ongoing
16
1L: Paclitaxel
2L: Adriamycin + Cyclophosphamide
3L: Capecitabine
4L: Sacituzumab govitecan
69 days, Ongoing
17
Lazaratos, Dankner et al. Supplemental Figure S1

## Slide 3
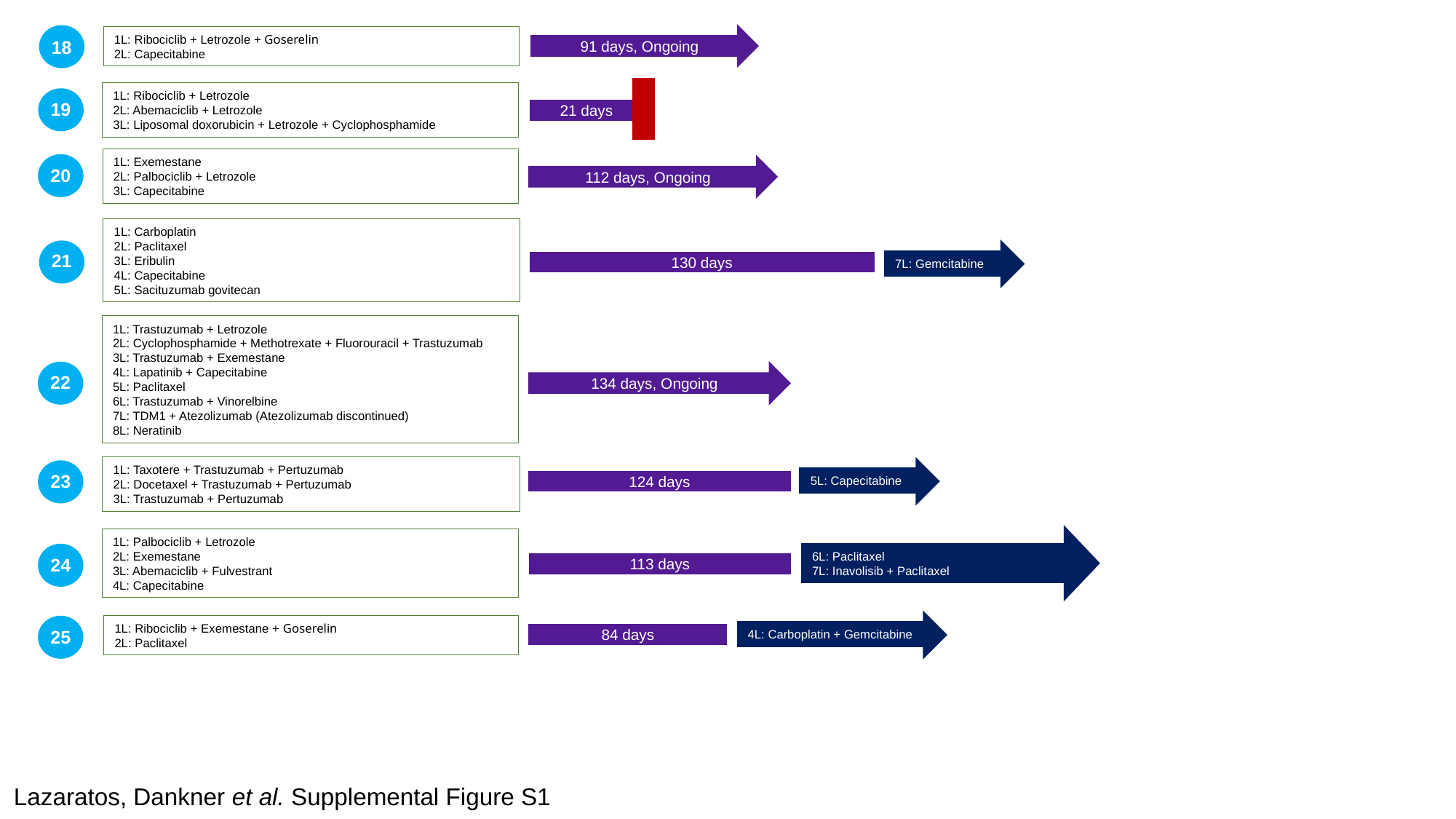

91 days, Ongoing
1L: Ribociclib + Letrozole + Goserelin
2L: Capecitabine
18
1L: Ribociclib + Letrozole
2L: Abemaciclib + Letrozole
3L: Liposomal doxorubicin + Letrozole + Cyclophosphamide
19
21 days
1L: Exemestane
2L: Palbociclib + Letrozole
3L: Capecitabine
112 days, Ongoing
20
1L: Carboplatin
2L: Paclitaxel
3L: Eribulin
4L: Capecitabine
5L: Sacituzumab govitecan
21
7L: Gemcitabine
130 days
1L: Trastuzumab + Letrozole
2L: Cyclophosphamide + Methotrexate + Fluorouracil + Trastuzumab
3L: Trastuzumab + Exemestane
4L: Lapatinib + Capecitabine
5L: Paclitaxel
6L: Trastuzumab + Vinorelbine
7L: TDM1 + Atezolizumab (Atezolizumab discontinued)
8L: Neratinib
134 days, Ongoing
22
1L: Taxotere + Trastuzumab + Pertuzumab
2L: Docetaxel + Trastuzumab + Pertuzumab
3L: Trastuzumab + Pertuzumab
23
5L: Capecitabine
124 days
1L: Palbociclib + Letrozole
2L: Exemestane
3L: Abemaciclib + Fulvestrant
4L: Capecitabine
6L: Paclitaxel
7L: Inavolisib + Paclitaxel
24
113 days
1L: Ribociclib + Exemestane + Goserelin
2L: Paclitaxel
25
4L: Carboplatin + Gemcitabine
84 days
Lazaratos, Dankner et al. Supplemental Figure S1

## Slide 4
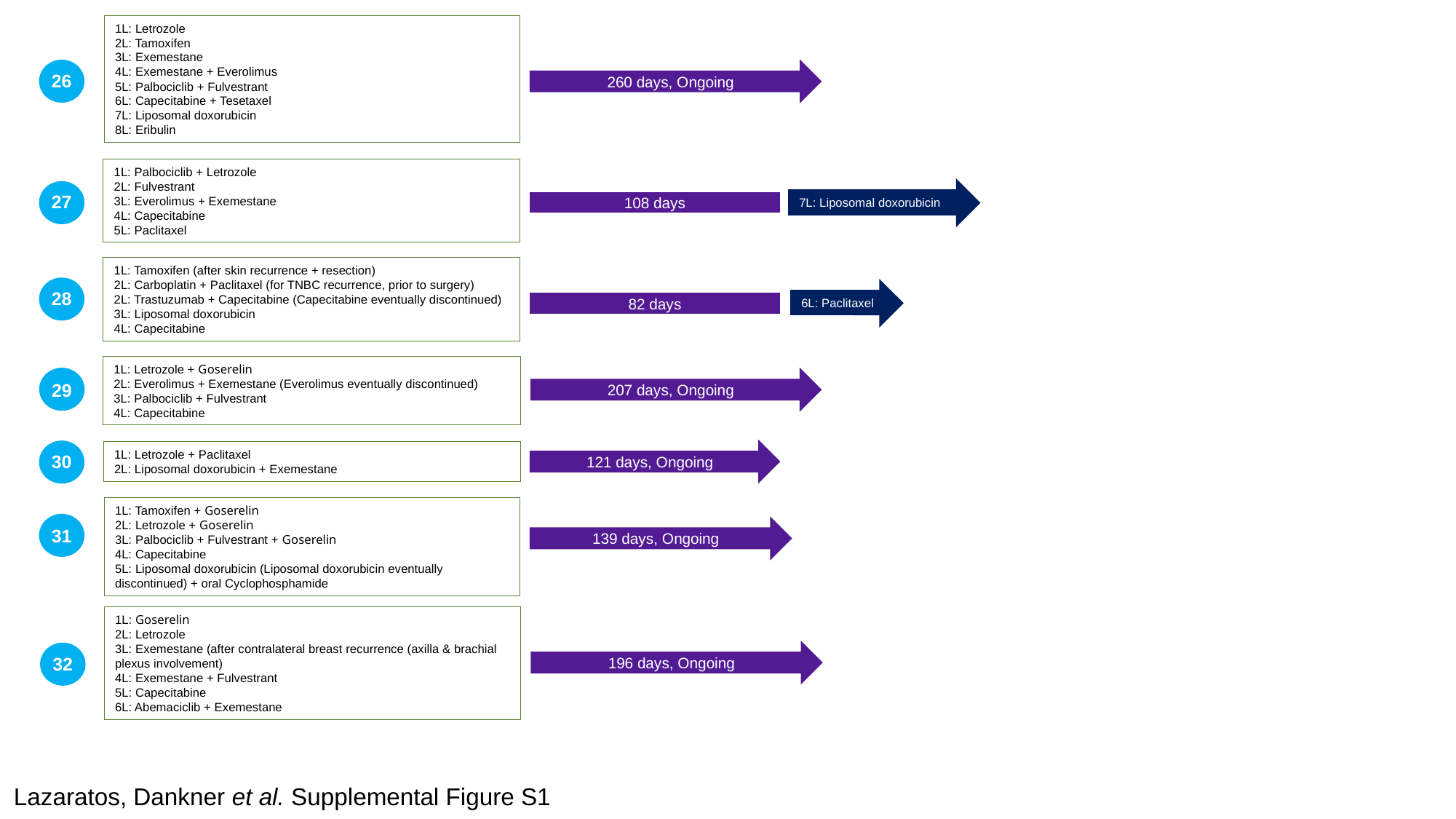

1L: Letrozole
2L: Tamoxifen
3L: Exemestane
4L: Exemestane + Everolimus
5L: Palbociclib + Fulvestrant
6L: Capecitabine + Tesetaxel
7L: Liposomal doxorubicin
8L: Eribulin
260 days, Ongoing
26
1L: Palbociclib + Letrozole
2L: Fulvestrant
3L: Everolimus + Exemestane
4L: Capecitabine
5L: Paclitaxel
27
7L: Liposomal doxorubicin
108 days
1L: Tamoxifen (after skin recurrence + resection)
2L: Carboplatin + Paclitaxel (for TNBC recurrence, prior to surgery)
2L: Trastuzumab + Capecitabine (Capecitabine eventually discontinued)
3L: Liposomal doxorubicin
4L: Capecitabine
28
6L: Paclitaxel
82 days
1L: Letrozole + Goserelin
2L: Everolimus + Exemestane (Everolimus eventually discontinued)
3L: Palbociclib + Fulvestrant
4L: Capecitabine
207 days, Ongoing
29
121 days, Ongoing
1L: Letrozole + Paclitaxel
2L: Liposomal doxorubicin + Exemestane
30
1L: Tamoxifen + Goserelin
2L: Letrozole + Goserelin
3L: Palbociclib + Fulvestrant + Goserelin
4L: Capecitabine
5L: Liposomal doxorubicin (Liposomal doxorubicin eventually discontinued) + oral Cyclophosphamide
139 days, Ongoing
31
1L: Goserelin
2L: Letrozole
3L: Exemestane (after contralateral breast recurrence (axilla & brachial plexus involvement)
4L: Exemestane + Fulvestrant
5L: Capecitabine
6L: Abemaciclib + Exemestane
196 days, Ongoing
32
Lazaratos, Dankner et al. Supplemental Figure S1

## Slide 5
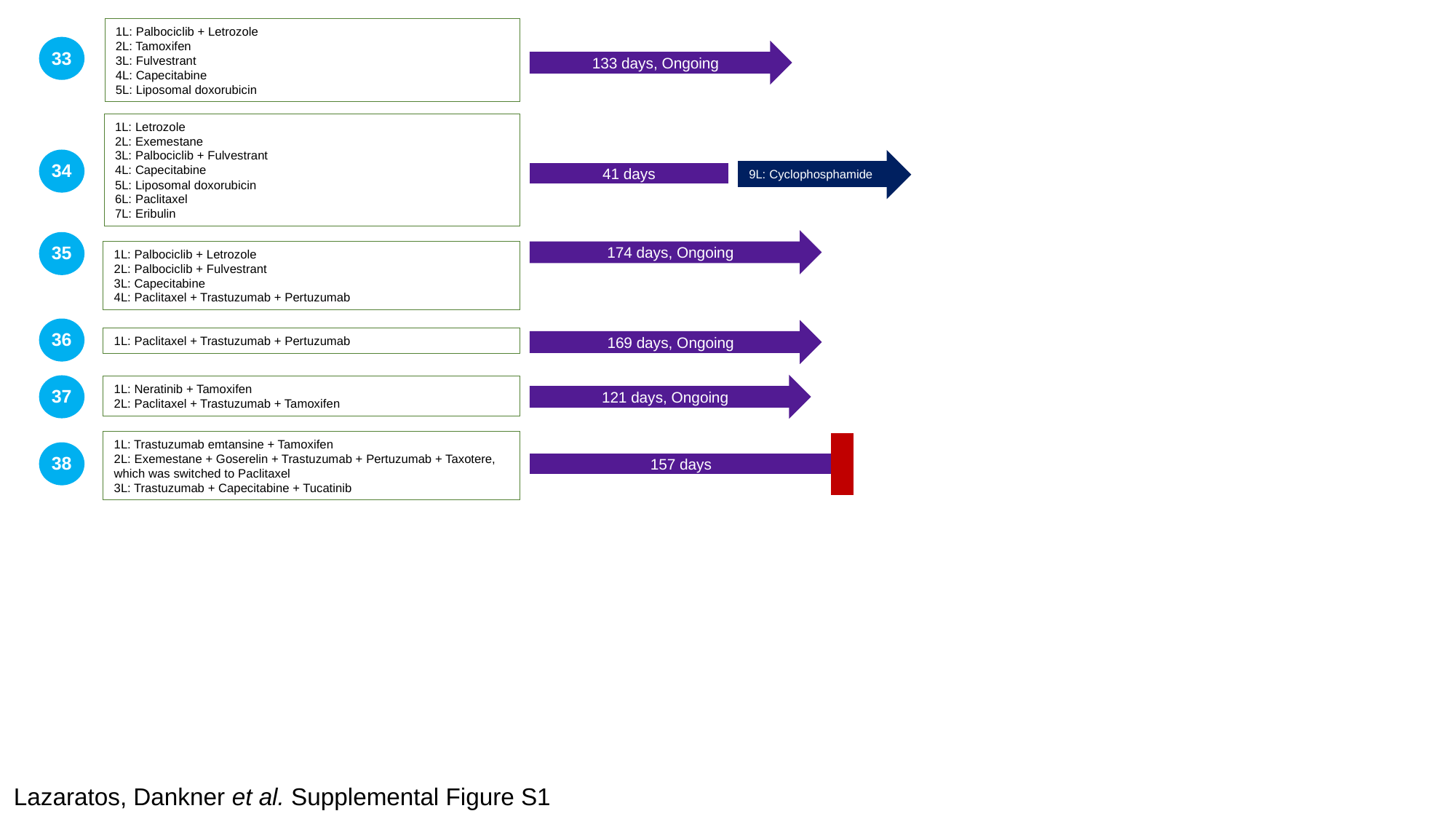

1L: Palbociclib + Letrozole
2L: Tamoxifen
3L: Fulvestrant
4L: Capecitabine
5L: Liposomal doxorubicin
33
133 days, Ongoing
1L: Letrozole
2L: Exemestane
3L: Palbociclib + Fulvestrant
4L: Capecitabine
5L: Liposomal doxorubicin
6L: Paclitaxel
7L: Eribulin
34
9L: Cyclophosphamide
41 days
174 days, Ongoing
35
1L: Palbociclib + Letrozole
2L: Palbociclib + Fulvestrant
3L: Capecitabine
4L: Paclitaxel + Trastuzumab + Pertuzumab
169 days, Ongoing
36
1L: Paclitaxel + Trastuzumab + Pertuzumab
1L: Neratinib + Tamoxifen
2L: Paclitaxel + Trastuzumab + Tamoxifen
121 days, Ongoing
37
1L: Trastuzumab emtansine + Tamoxifen
2L: Exemestane + Goserelin + Trastuzumab + Pertuzumab + Taxotere, which was switched to Paclitaxel
3L: Trastuzumab + Capecitabine + Tucatinib
38
157 days
Lazaratos, Dankner et al. Supplemental Figure S1
