# Supplementary material for: The Real-World Clinical Outcomes of Heavily Pretreated HER2+ and HER2-Low Metastatic Breast Cancer Patients Treated with Trastuzumab Deruxtecan at a Single Centre
Source: Curr Oncol. 2024 Dec 24;32(1):1. doi: 10.3390/curroncol32010001 (PMC11763754; doi:10.3390/curroncol32010001)
Supplement: Supplementary file 1 [file curroncol-32-00001-s001.zip › Supplemental Figure S2.pptx]

## Slide 1
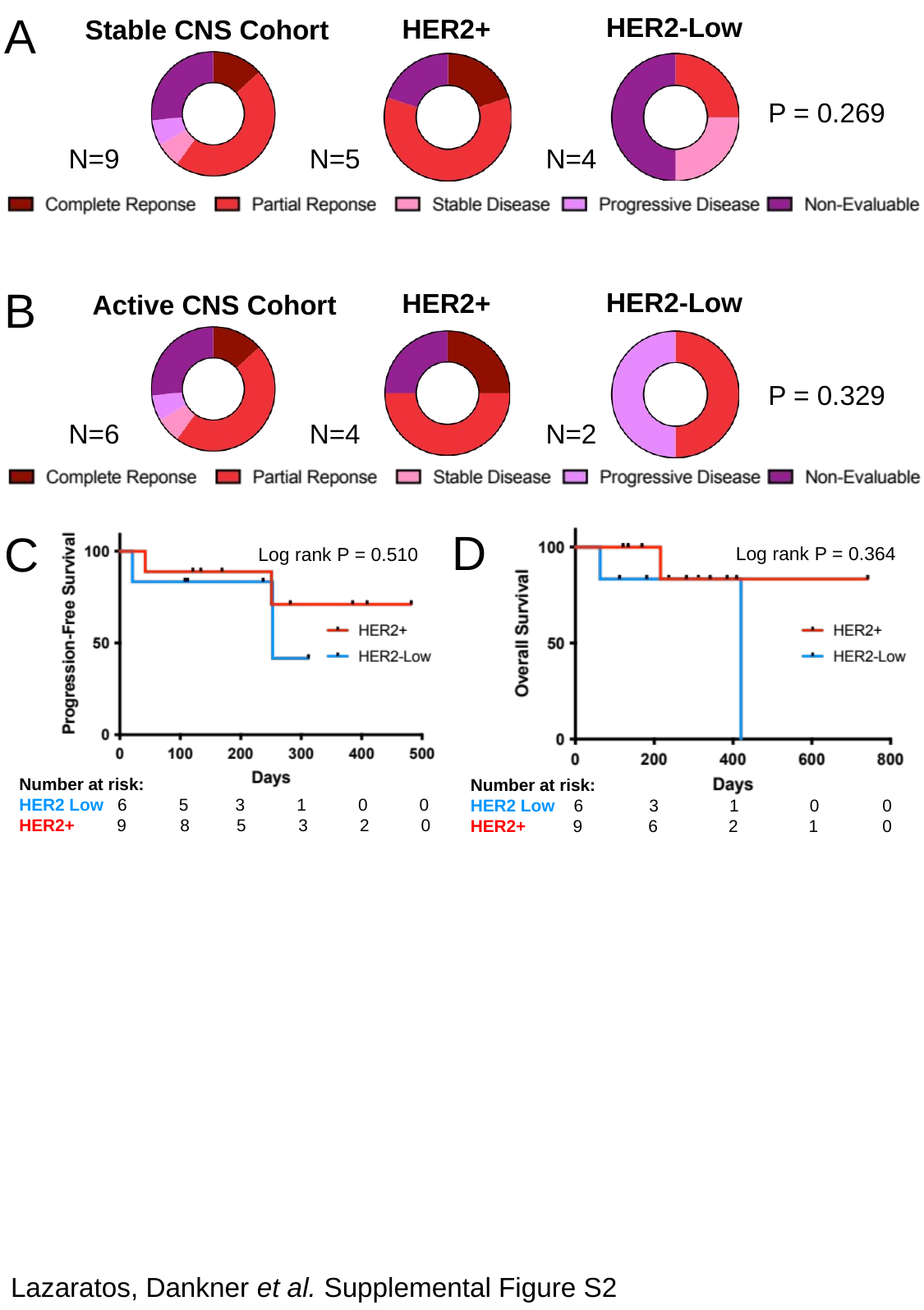

A
HER2-Low
HER2+
Stable CNS Cohort
P = 0.269
N=9
N=5
N=4
B
HER2-Low
HER2+
Active CNS Cohort
P = 0.329
N=6
N=4
N=2
D
C
Log rank P = 0.364
Log rank P = 0.510
Number at risk:
HER2 Low 6 5 3 1 0 0
HER2+ 9	 8 5	 3 2 0
Number at risk:
HER2 Low 6 3 1 0	 0
HER2+ 9 6 2 1	 0
Lazaratos, Dankner et al. Supplemental Figure S2

## Slide 2
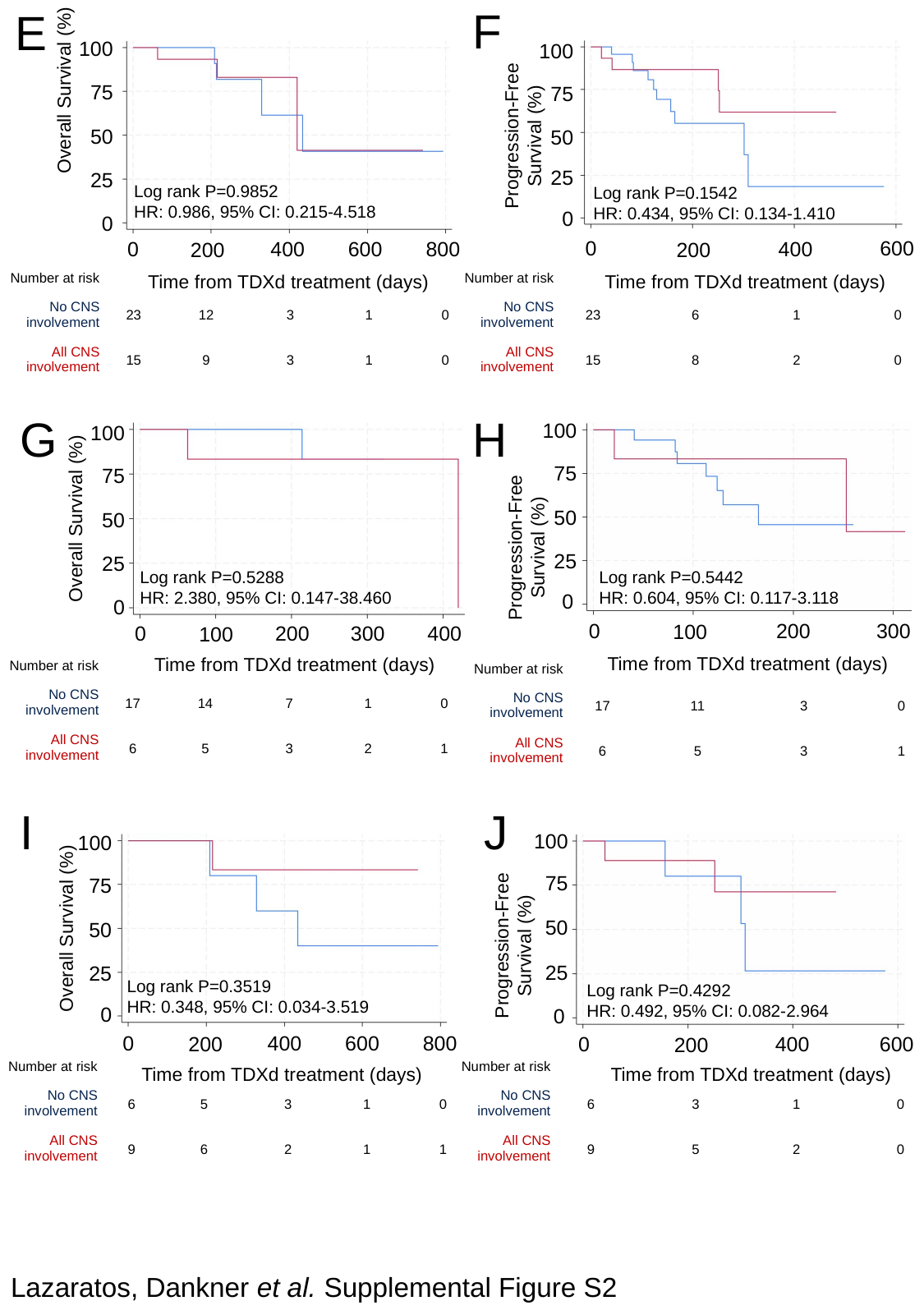

F
E
100
100
Overall Survival (%)
75
75
Progression-Free Survival (%)
50
50
25
25
Log rank P=0.9852
HR: 0.986, 95% CI: 0.215-4.518
Log rank P=0.1542
HR: 0.434, 95% CI: 0.134-1.410
0
0
0
600
400
0
600
800
400
200
200
Time from TDXd treatment (days)
Time from TDXd treatment (days)
| Number at risk | | | | | |
| --- | --- | --- | --- | --- | --- |
| No CNS involvement | 23 | 12 | 3 | 1 | 0 |
| All CNS involvement | 15 | 9 | 3 | 1 | 0 |
| Number at risk | | | | |
| --- | --- | --- | --- | --- |
| No CNS involvement | 23 | 6 | 1 | 0 |
| All CNS involvement | 15 | 8 | 2 | 0 |
G
H
100
100
75
75
50
Overall Survival (%)
50
Progression-Free Survival (%)
25
25
Log rank P=0.5288
HR: 2.380, 95% CI: 0.147-38.460
Log rank P=0.5442
HR: 0.604, 95% CI: 0.117-3.118
0
0
0
300
200
100
0
300
400
200
100
Time from TDXd treatment (days)
Time from TDXd treatment (days)
| Number at risk | | | | | |
| --- | --- | --- | --- | --- | --- |
| No CNS involvement | 17 | 14 | 7 | 1 | 0 |
| All CNS involvement | 6 | 5 | 3 | 2 | 1 |
| Number at risk | | | | |
| --- | --- | --- | --- | --- |
| No CNS involvement | 17 | 11 | 3 | 0 |
| All CNS involvement | 6 | 5 | 3 | 1 |
I
J
100
100
75
75
50
Overall Survival (%)
50
Progression-Free Survival (%)
25
25
Log rank P=0.3519
HR: 0.348, 95% CI: 0.034-3.519
Log rank P=0.4292
HR: 0.492, 95% CI: 0.082-2.964
0
0
0
600
800
400
0
600
400
200
200
| Number at risk | | | | | |
| --- | --- | --- | --- | --- | --- |
| No CNS involvement | 6 | 5 | 3 | 1 | 0 |
| All CNS involvement | 9 | 6 | 2 | 1 | 1 |
| Number at risk | | | | |
| --- | --- | --- | --- | --- |
| No CNS involvement | 6 | 3 | 1 | 0 |
| All CNS involvement | 9 | 5 | 2 | 0 |
Time from TDXd treatment (days)
Time from TDXd treatment (days)
Lazaratos, Dankner et al. Supplemental Figure S2

## Slide 3
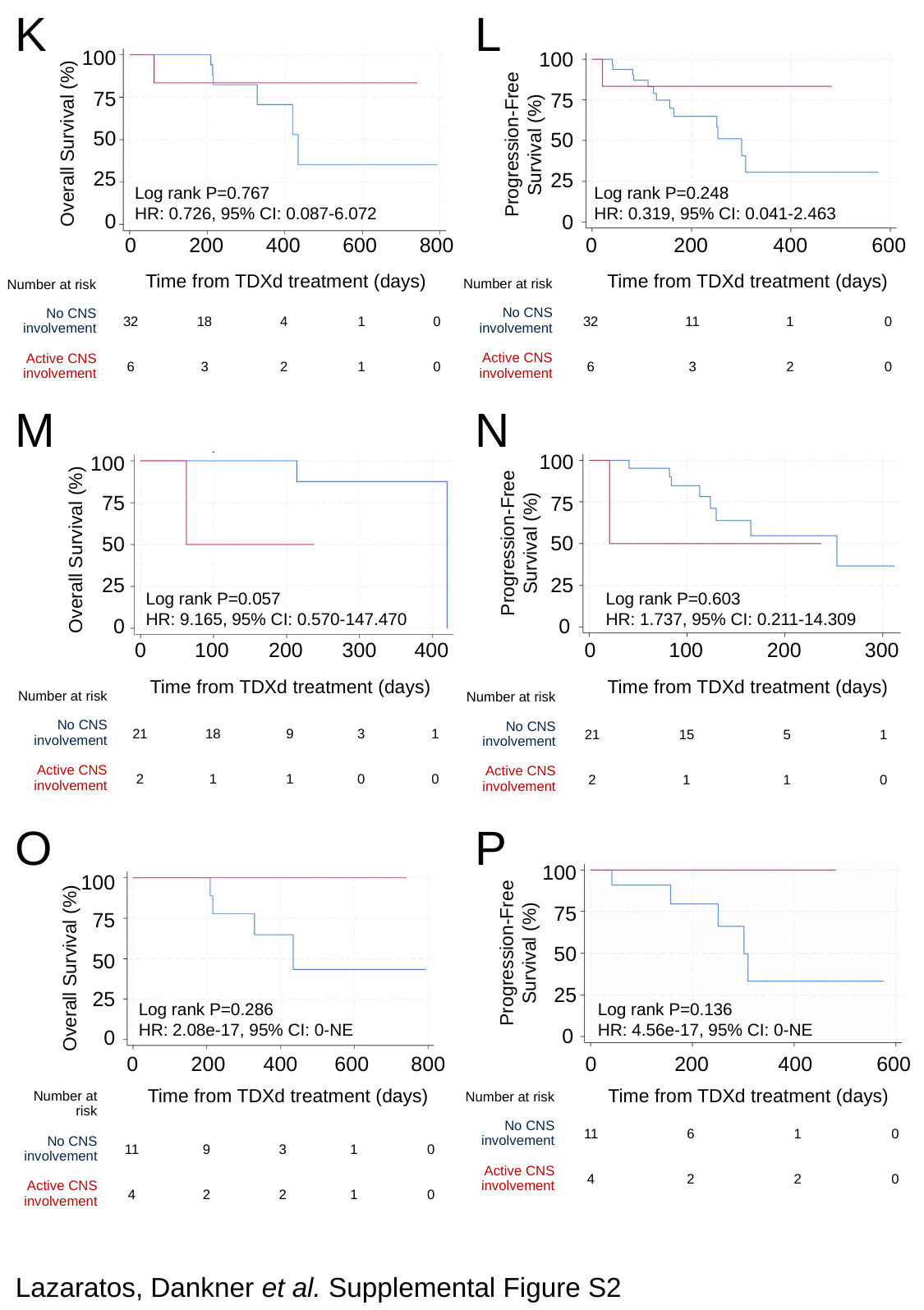

K
L
100
100
75
75
Progression-Free Survival (%)
50
50
Overall Survival (%)
25
25
Log rank P=0.767
HR: 0.726, 95% CI: 0.087-6.072
Log rank P=0.248
HR: 0.319, 95% CI: 0.041-2.463
0
0
0
200
400
600
800
0
200
400
600
Time from TDXd treatment (days)
Time from TDXd treatment (days)
| Number at risk | | | | |
| --- | --- | --- | --- | --- |
| No CNS involvement | 32 | 11 | 1 | 0 |
| Active CNS involvement | 6 | 3 | 2 | 0 |
| Number at risk | | | | | |
| --- | --- | --- | --- | --- | --- |
| No CNS involvement | 32 | 18 | 4 | 1 | 0 |
| Active CNS involvement | 6 | 3 | 2 | 1 | 0 |
M
N
100
100
75
75
Progression-Free Survival (%)
50
50
Overall Survival (%)
25
25
Log rank P=0.057
HR: 9.165, 95% CI: 0.570-147.470
Log rank P=0.603
HR: 1.737, 95% CI: 0.211-14.309
0
0
0
100
200
300
400
0
100
200
300
Time from TDXd treatment (days)
Time from TDXd treatment (days)
| Number at risk | | | | | |
| --- | --- | --- | --- | --- | --- |
| No CNS involvement | 21 | 18 | 9 | 3 | 1 |
| Active CNS involvement | 2 | 1 | 1 | 0 | 0 |
| Number at risk | | | | |
| --- | --- | --- | --- | --- |
| No CNS involvement | 21 | 15 | 5 | 1 |
| Active CNS involvement | 2 | 1 | 1 | 0 |
O
P
100
100
75
75
Progression-Free Survival (%)
50
50
Overall Survival (%)
25
25
Log rank P=0.286
HR: 2.08e-17, 95% CI: 0-NE
Log rank P=0.136
HR: 4.56e-17, 95% CI: 0-NE
0
0
0
200
400
600
800
0
200
400
600
Time from TDXd treatment (days)
Time from TDXd treatment (days)
| Number at risk | | | | | |
| --- | --- | --- | --- | --- | --- |
| No CNS involvement | 11 | 9 | 3 | 1 | 0 |
| Active CNS involvement | 4 | 2 | 2 | 1 | 0 |
| Number at risk | | | | |
| --- | --- | --- | --- | --- |
| No CNS involvement | 11 | 6 | 1 | 0 |
| Active CNS involvement | 4 | 2 | 2 | 0 |
Lazaratos, Dankner et al. Supplemental Figure S2

## Slide 4
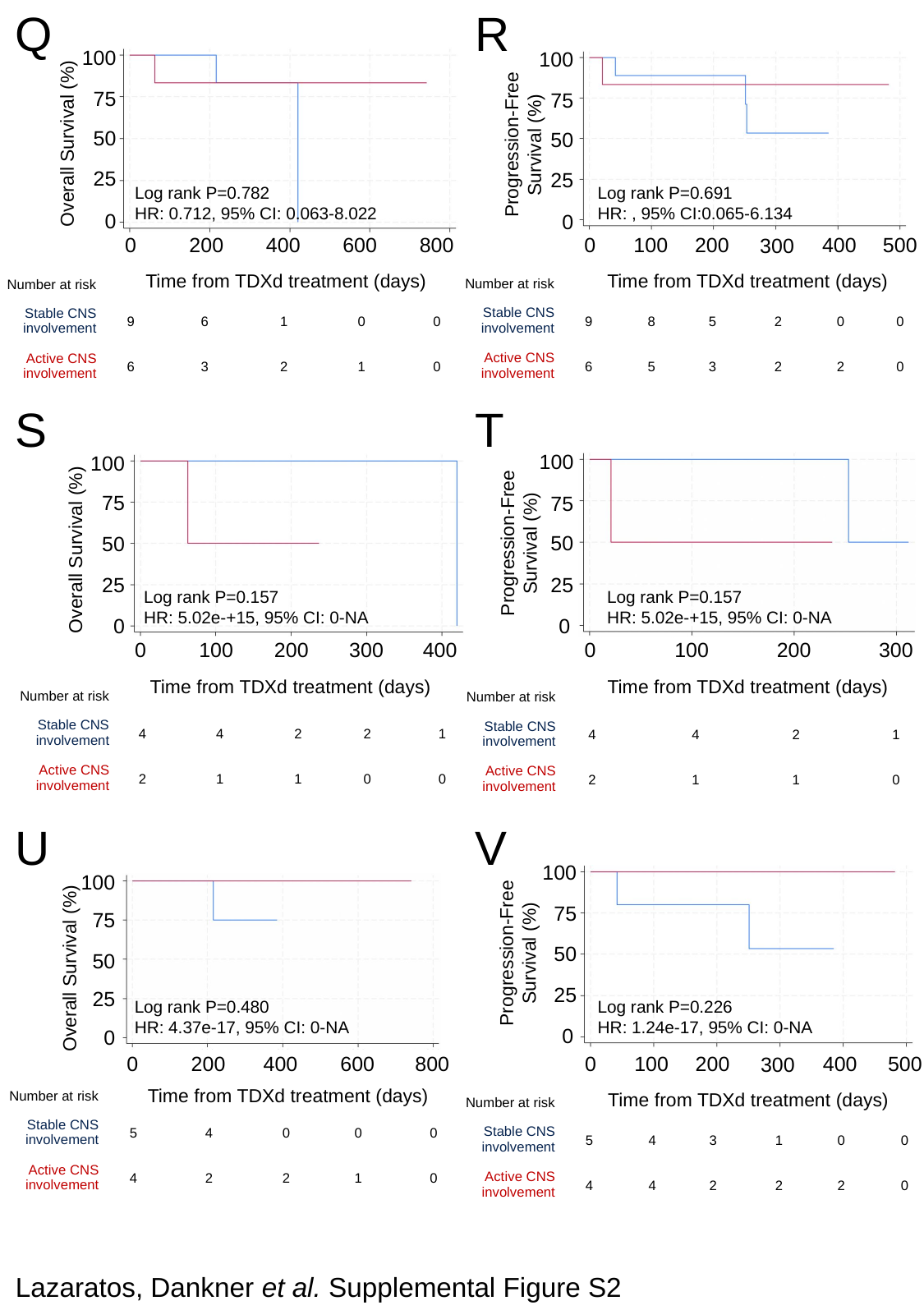

Q
R
100
100
75
75
Progression-Free Survival (%)
50
50
Overall Survival (%)
25
25
Log rank P=0.782
HR: 0.712, 95% CI: 0.063-8.022
Log rank P=0.691
HR: , 95% CI:0.065-6.134
0
0
100
0
200
400
600
800
0
200
400
500
300
Time from TDXd treatment (days)
Time from TDXd treatment (days)
| Number at risk | | | | | | |
| --- | --- | --- | --- | --- | --- | --- |
| Stable CNS involvement | 9 | 8 | 5 | 2 | 0 | 0 |
| Active CNS involvement | 6 | 5 | 3 | 2 | 2 | 0 |
| Number at risk | | | | | |
| --- | --- | --- | --- | --- | --- |
| Stable CNS involvement | 9 | 6 | 1 | 0 | 0 |
| Active CNS involvement | 6 | 3 | 2 | 1 | 0 |
S
T
100
100
75
75
Progression-Free Survival (%)
50
50
Overall Survival (%)
25
25
Log rank P=0.157
HR: 5.02e-+15, 95% CI: 0-NA
Log rank P=0.157
HR: 5.02e-+15, 95% CI: 0-NA
0
0
0
100
200
300
400
0
100
200
300
Time from TDXd treatment (days)
Time from TDXd treatment (days)
| Number at risk | | | | | |
| --- | --- | --- | --- | --- | --- |
| Stable CNS involvement | 4 | 4 | 2 | 2 | 1 |
| Active CNS involvement | 2 | 1 | 1 | 0 | 0 |
| Number at risk | | | | |
| --- | --- | --- | --- | --- |
| Stable CNS involvement | 4 | 4 | 2 | 1 |
| Active CNS involvement | 2 | 1 | 1 | 0 |
U
V
100
100
75
75
Progression-Free Survival (%)
50
50
Overall Survival (%)
25
25
Log rank P=0.480
HR: 4.37e-17, 95% CI: 0-NA
Log rank P=0.226
HR: 1.24e-17, 95% CI: 0-NA
0
0
100
0
200
400
600
800
0
200
400
500
300
Time from TDXd treatment (days)
Time from TDXd treatment (days)
| Number at risk | | | | | |
| --- | --- | --- | --- | --- | --- |
| Stable CNS involvement | 5 | 4 | 0 | 0 | 0 |
| Active CNS involvement | 4 | 2 | 2 | 1 | 0 |
| Number at risk | | | | | | |
| --- | --- | --- | --- | --- | --- | --- |
| Stable CNS involvement | 5 | 4 | 3 | 1 | 0 | 0 |
| Active CNS involvement | 4 | 4 | 2 | 2 | 2 | 0 |
Lazaratos, Dankner et al. Supplemental Figure S2
